# Associations between genetic polymorphisms of TLRs and susceptibility to tuberculosis: A meta-analysis

**DOI:** 10.1177/1753425919862354

**Published:** 2019-07-18

**Authors:** Yong Zhou, Mengtao Zhang

**Affiliations:** 1 Endoscope Center, Xi’an Thoracic Hospital, PR China; 2 Medical Department, Xi’an Thoracic Hospital, PR China

**Keywords:** TLR, gene polymorphisms, tuberculosis (TB), meta-analysis, ethnicities

## Abstract

Some genetic association studies have tried to investigate potential associations between TLR polymorphisms and tuberculosis. However, the results of these studies have not been consistent. Thus, we performed the present meta-analysis to explore associations between TLR polymorphisms and tuberculosis in a larger combined population. A systematic literature research of PubMed, Web of Science and Embase was performed to identify eligible studies for combined analyses. *I*^2^ statistics were employed to assess between-study heterogeneities. If *I*^2^ was >50%, random-effects models were used to combine the data. Otherwise, fixed-effects models were applied for synthetic analyses. A total of 39 genetic association studies were included in the analyses. The combined analyses showed that *TLR1* rs4833095, *TLR1* rs5743557, *TLR1* rs5743596, *TLR2* rs3804099, *TLR2* rs5743704, *TLR2* rs5743708, *TLR6* rs5743810 and *TLR8* rs3764879 polymorphisms were significantly associated with susceptibility to TB in the overall population. Further subgroup analyses revealed similar significant findings for *TLR1* rs4833095, *TLR1* rs5743557, *TLR1* rs5743596, *TLR1* rs5743618, *TLR2* rs3804099, *TLR2* rs5743704, *TLR2* rs5743708, *TLR4* rs4986790 and *TLR4* rs4986791 polymorphisms in certain ethnicities. In conclusion, our findings support that these *TLR* polymorphisms may be used to identify individuals at high risk of developing tuberculosis.

## Introduction

Tuberculosis (TB) is a common chronic infectious disorder caused by *Mycobacterium tuberculosis* (MTB), and it could manifest as pulmonary tuberculosis and/or extrapulmonary tuberculosis.^1^ Despite rapid advancements achieved in early diagnosis and pharmacological therapy over the past few decades, TB remains a serious public-health threat. According to a recent epidemiological study, about 30% of the general population is currently infected with MTB, and around 5–10% of these infected individuals will eventually develop active TB.^2^ The course of MTB infection depends on a complex interaction of pathogen, host and environmental factors, and the fact that only a small portion of infected individuals eventually develop active TB suggests that host genetic background is crucial for its development.^3,4^

TLRs are a group of type 1 transmembrane proteins expressed on a variety of immune cells that recognise stimuli from exogenous pathogens.^5,6^ The binding of TLRs with their corresponding ligands leads to recruitment of adaptor proteins, activation of downstream signal transduction pathways, up-regulation of cytokine and chemokine production, and ultimately the development of immune responses against exogenous pathogens.^7,8^ Consequently, it is possible that *TLR* gene polymorphisms, which may impact biological activities of TLRs, might also be involved in the development of multiple infectious diseases, including TB.^9^

To date, numerous studies have already investigated potential associations between *TLR* gene polymorphisms and TB. However, the results of these studies were not consistent, especially when they were conducted in different populations. Previous studies failed to reach a consensus regarding associations between *TLR* gene polymorphisms and TB, in part because of their relatively small sample sizes. Thus, we performed the present meta-analysis to explore the relationship between *TLR* gene polymorphisms and TB in a larger combined population. In addition, we also aimed to elucidate the potential effects of ethnic background on associations between *TLR* gene polymorphisms and TB.

## Materials and methods

The current meta-analysis was reported according to the Preferred Reporting Items for Systematic Reviews and Meta-Analyses statement.^10^

### Literature search and inclusion criteria

Potentially relevant articles were searched in PubMed, Medline and Web of Science using the following keywords: ‘Toll like receptor’, ‘TLR’, ‘polymorphism’, ‘variant’, ‘mutation’, ‘SNP’, ‘variation’, ‘genotype’, ‘allele’, ‘tuberculosis’ and ‘TB’. The initial literature search was performed in January 2019, and the latest update was finished in May 2019. Moreover, we also screened the references of all retrieved articles to identify other potential relevant studies.

Inclusion criteria were (a) genetic association studies on associations between *TLR* gene polymorphisms and TB in human beings, (b) genotypic frequency of investigated *TLR* gene polymorphisms provided in cases and controls and (c) full text available in English. For duplicate reports, only the most complete one was included. Excluded criteria were (a) not about *TLR* gene polymorphisms and TB, (b) not performed on human beings, (c) case reports or case series and (d) reviews, comments and conference presentations.

### Data extraction and quality assessment

The following data were extracted from the included studies: (a) last name of first author, (b) year of publication, (c) country where the study was conducted and ethnicity of study participants, (d) type of disease, (e) the number of cases and controls and (f) genotypic distributions of *TLR* gene polymorphisms in cases and controls. The P-value for Hardy–Weinberg equilibrium (HWE) was also calculated. When necessary, we wrote to the corresponding authors for extra information. We used the Newcastle–Ottawa scale (NOS) to assess the quality of eligible studies.^11^ This scale has a score range of zero to nine, and studies with a score of more than seven were thought to be of high quality. Data extraction and quality assessment were performed by two independent reviewers. Any disagreement between two reviewers was solved by discussion until a consensus was reached.

### Statistical analyses

We used Review Manager v5.3.3 (The Cochrane Collaboration, London, UK) to conduct statistical analyses. We calculated odds ratios (ORs) and 95% confidence intervals (CIs) to estimate the strength of associations between *TLR* gene polymorphisms and TB in dominant, recessive, over-dominant and allele models. Statistical significances of combined analyses were determined by the *Z*-test, with a **P-value of ≤ 0.05 defined as statistically significant. *I*^2^ statistics were employed to assess between-study heterogeneities. If *I*^2^ was >50%, random-effects models (REMs; DerSimonian–Laird method) was used to combine the data because of significant heterogeneities. Otherwise, fixed-effects models (Mantel–Haenszel method) were used for synthetic analyses. Subgroup analyses by ethnicity of participants were subsequently performed to evaluate effects of ethnic background on investigated genetic associations. Sensitivity analyses were carried out to test the stability of combined results by omitting one study at a time and performing the analyses again based on the results of the remaining studies. Publication biases were evaluated with funnel plots.

## Results

### Characteristics of included studies

The initial literature search identified 573 potential relevant articles. After exclusion of irrelevant and duplicate articles by reading titles and abstracts, 78 potentially relevant articles were retrieved for eligibility assessment. Another 39 articles were subsequently excluded after reading the full text. Finally, 39 studies that met the inclusion criteria were included (see online supplemental Figure S1). Baseline characteristics of included studies are shown in Table 1. The full manuscripts of the included studies can be found at Open Science Framework (https://osf.io). Data sets are also available to readers upon request.

**Table 1.** The characteristics of 39 included studies for this meta-analysis.

First author, Yr	Country	Ethnicity	Type of disease	Samplesize	Genotypes (wtwt/wtmt/mtmt)		P-value for HWE	NOS score
					Cases	Controls		
*TLR1 rs4833095*					CC/CT/TT			
Dittrich, 2015	Germany	Caucasian	TB	206/239	42/99/65	74/108/57	0.157	7
Kobayashi, 2012	Indonesia	South Asian	PTB	533/557	186/258/89	216/250/91	0.196	8
Ma, 2007	USA	African	TB	339/194	240/68/31	116/61/17	0.037	7
Ma, 2007	USA	Caucasian	TB	555/224	239/215/101	114/83/27	0.057	7
Peng, 2017	PR China	East Asian	TB	646/475	240/304/102	174/212/89	0.090	7
Qi, 2015	PR China	East Asian	TB	340/366	154/136/50	149/168/49	0.880	8
Salie, 2015	South Africa	African	TB	324/344	166/123/35	168/143/33	0.749	7
Sinha, 2014	India	South Asian	PTB	205/127	53/97/55	29/78/20	0.008	7
Zhang, 2018	PR China	East Asian	TB	613/603	230/280/103	221/298/84	0.300	7
Zhang, 2019	PR China	East Asian	TB	409/204	145/189/75	56/116/32	0.029	7
*TLR1 rs5743557*					GG/GA/AA			
Peng, 2017	PR China	East Asian	TB	646/475	230/300/116	134/248/93	0.257	7
Qi, 2015	PR China	East Asian	TB	340/366	107/152/81	95/177/94	0.531	8
Zhang, 2018	PR China	East Asian	TB	613/602	315/251/47	254/259/89	0.087	7
Zhang, 2019	PR China	East Asian	TB	409/204	131/210/68	64/114/26	0.024	7
*TLR1 rs5743596*					GG/GA/AA			
Peng, 2017	PR China	East Asian	TB	646/475	320/262/64	223/207/45	0.761	7
Qi, 2015	PR China	East Asian	TB	340/366	132/144/64	143/161/62	0.152	8
Zhang, 2018	PR China	East Asian	TB	613/602	370/212/31	313/240/49	0.753	7
Zhang, 2019	PR China	East Asian	TB	409/204	190/179/40	88/98/18	0.204	7
*TLR1 rs5743604*					GG/GA/AA			
Kobayashi, 2012	Indonesia	South Asian	PTB	534/558	134/272/128	162/253/143	0.030	8
Qi, 2015	PR China	East Asian	TB	340/366	120/154/66	115/184/67	0.659	8
Zhang, 2018	PR China	East Asian	TB	613/602	120/303/190	156/291/155	0.415	7
Zhang, 2019	PR China	East Asian	TB	409/204	106/210/93	46/115/43	0.068	7
*TLR1 rs5743618*					TT/TG/GG			
Barletta-Naveca, 2018	Brazil	Mixed	PTB	252/210	146/86/20	116/74/20	0.114	7
Ma, 2007	USA	African	TB	339/194	272/63/4	120/61/13	0.180	7
Ma, 2007	USA	Caucasian	TB	555/224	379/144/32	124/72/28	0.001	7
Ma, 2010	PR China	East Asian	PTB	543/544	510/32/1	509/34/1	0.588	8
Naderi, 2016	Iran	South Asian	PTB	203/203	156/47/0	186/17/0	0.534	7
Ocejo-Vinyals, 2013	Spain	Caucasian	PTB	190/192	50/82/58	60/98/34	0.580	8
Qi, 2015	PR China	East Asian	TB	340/366	295/45/0	345/21/0	0.572	8
Salie, 2015	South Africa	African	TB	328/330	235/90/3	244/79/7	0.839	7
Selvaraj, 2010	India	South Asian	PTB	202/205	192/9/1	189/16/0	0.561	8
Sinha, 2014	India	South Asian	PTB	160/124	140/20/0	100/23/1	0.797	7
Wu, 2015	PR China	East Asian	TB	334/422	298/33/3	350/70/2	0.449	8
*TLR2 rs3804099*					TT/TC/CC			
Arji, 2014	Morocco	Caucasian	PTB	343/202	100/169/74	50/121/31	0.003	7
Caws, 2008	Vietnam	Mixed	PTB	165/377	87/67/11	205/154/18	0.105	7
Caws, 2008	Vietnam	Mixed	EPTB	141/377	66/55/20	205/154/18	0.105	7
Etokebe, 2010	Norway	Caucasian	TB	97/102	34/47/16	38/50/14	0.702	7
Kobayashi, 2012	Indonesia	South Asian	PTB	538/558	377/145/16	359/183/16	0.200	8
Salie, 2015	South Africa	African	TB	435/292	146/214/75	91/143/58	0.893	7
Sánchez, 2012	Colombia	Mixed	PTB	465/300	173/220/72	95/153/52	0.473	7
Torres-García, 2013	Mexico	Mixed	PTB	90/90	59/26/5	48/36/6	0.829	8
Varzari, 2019	Germany	Caucasian	PTB	115/145	54/49/12	40/76/29	0.513	7
Wu, 2015	PR China	East Asian	TB	334/422	169/131/34	191/180/51	0.395	8
Yang, 2013	PR China	East Asian	PTB	200/196	97/83/20	97/81/18	0.854	7
Zhang, 2018	PR China	East Asian	TB	321/475	176/130/15	243/187/45	0.305	8
Zhao, 2015	PR China	East Asian	PTB	230/386	104/94/32	166/183/37	0.185	8
Zhao, 2015	PR China	East Asian	EPTB	111/386	53/53/5	166/183/37	0.185	8
*TLR2 rs3804100*					TT/TC/CC			
Chen, 2010	Taiwan	East Asian	PTB	184/184	131/45/8	121/55/8	0.586	7
Etokebe, 2010	Norway	Caucasian	TB	97/105	81/15/1	89/16/0	0.398	7
Kobayashi, 2012	Indonesia	South Asian	PTB	533/559	411/111/11	413/126/20	0.010	8
Salie, 2015	South Africa	African	TB	435/292	391/44/0	244/48/0	0.126	7
Wu, 2015	PR China	East Asian	TB	334/422	134/134/66	212/168/42	0.309	8
Zhang, 2018	PR China	East Asian	TB	634/475	358/233/43	267/172/36	0.262	8
*TLR2 rs5743704*					CC/CA/AA			
Etokebe, 2010	Norway	Caucasian	TB	103/105	93/10/0	101/4/0	0.842	7
Panwar, 2016	India	South Asian	PTB	106/106	105/1/0	106/0/0	NA	8
Panwar, 2016	India	South Asian	EPTB	106/106	101/5/0	106/0/0	NA	8
Rizvi, 2016	India	South Asian	PTB	130/130	129/1/0	130/0/0	NA	8
Rizvi, 2016	India	South Asian	EPTB	130/130	125/5/0	130/0/0	NA	8
Salie, 2015	South Africa	African	TB	438/292	432/6/0	287/5/0	0.883	7
Sánchez, 2012	Colombia	Mixed	PTB	466/299	448/18/0	291/8/0	0.815	7
*TLR2 rs5743708*					GG/GA/AA			
Barletta-Naveca, 2018	Brazil	Mixed	PTB	196/168	196/0/0	168/0/0	NA	7
Dalgic, 2011	Turkey	Caucasian	TB	198/200	152/46/0	186/14/0	0.608	7
Etokebe, 2010	Norway	Caucasian	TB	103/105	102/1/0	104/1/0	0.961	7
Jafari, 2016	Iran	South Asian	PTB	96/122	96/0/0	120/2/0	0.927	7
Mittal, 2018	India	South Asian	PTB	155/98	154/1/0	98/0/0	NA	7
Olesen, 2007	Gambia	African	PTB	321/347	321/0/0	347/0/0	NA	8
Panwar, 2016	India	South Asian	PTB	106/106	105/1/0	106/0/0	NA	8
Panwar, 2016	India	South Asian	EPTB	106/106	104/2/0	106/0/0	NA	8
Rizvi, 2016	India	South Asian	PTB	130/130	129/1/0	130/0/0	NA	8
Rizvi, 2016	India	South Asian	EPTB	130/130	128/2/0	130/0/0	NA	8
Salie, 2015	South Africa	African	TB	438/288	426/12/0	284/4/0	0.906	7
Sánchez, 2012	Colombia	Mixed	PTB	466/300	463/3/0	296/4/0	0.907	7
Selvaraj, 2010	India	South Asian	PTB	193/199	192/1/0	198/1/0	0.972	8
Torres-García, 2013	Mexico	Mixed	PTB	90/90	90/0/0	90/0/0	NA	8
Wu, 2015	PR China	East Asian	TB	334/422	319/15/0	418/4/0	0.922	8
*TLR4 rs4986790*					AA/AG/GG			
Barletta-Naveca, 2018	Brazil	Mixed	PTB	238/208	221/16/1	199/8/1	0.009	7
Biyikli, 2016	Turkey	Caucasian	TB	29/100	28/1/0	96/4/0	0.838	7
Fitness, 2004	UK	Caucasian	PTB	282/427	258/24/0	389/38/0	0.336	7
Jafari, 2016	Iran	South Asian	PTB	96/122	82/14/0	115/7/0	0.744	7
Jahantigh, 2013	Iran	South Asian	PTB	124/149	122/2/0	146/3/0	0.901	8
Ma, 2007	USA	African	TB	339/194	281/57/1	157/36/1	0.484	7
Ma, 2007	USA	Caucasian	TB	555/224	512/42/1	201/22/1	0.638	7
Najmi, 2010	India	South Asian	PTB	135/250	95/34/6	206/44/0	0.127	7
Olesen, 2007	Gambia	African	PTB	315/337	262/51/2	265/65/7	0.212	8
Rosas-Taraco, 2007	Mexico	Mixed	PTB	104/114	94/10/0	110/4/0	0.849	8
Salie, 2015	South Africa	African	TB	421/287	374/47/0	264/23/0	0.479	7
Sánchez, 2012	Colombia	Mixed	PTB	466/300	429/36/1	270/29/1	0.814	7
Selvaraj, 2010	India	South Asian	PTB	204/207	153/47/4	151/53/3	0.493	8
Torres-García, 2013	Mexico	Mixed	PTB	90/90	88/2/0	89/1/0	0.958	8
Wang, 2017	PR China	East Asian	TB	310/622	163/120/27	359/221/42	0.318	7
Wu, 2015	PR China	East Asian	TB	334/422	258/73/3	346/75/1	0.140	8
*TLR4 rs4986791*					CC/CT/TT			
Barletta-Naveca, 2018	Brazil	Mixed	PTB	238/208	221/16/1	199/8/1	0.009	7
Biyikli, 2016	Turkey	Caucasian	TB	29/100	28/1/0	94/6/0	0.757	7
Jafari, 2016	Iran	South Asian	PTB	96/122	88/8/0	120/2/0	0.927	7
Jahantigh, 2013	Iran	South Asian	PTB	124/149	112/10/2	141/7/1	0.016	8
Ma, 2007	USA	African	TB	339/194	325/14/0	178/16/0	0.549	7
Ma, 2007	USA	Caucasian	TB	555/224	518/36/1	205/18/1	0.386	7
Najmi, 2010	India	South Asian	PTB	135/250	105/26/4	206/43/1	0.429	7
Salie, 2015	South Africa	African	TB	439/292	417/22/0	275/16/1	0.157	7
Sánchez, 2012	Colombia	Mixed	PTB	466/299	429/36/1	272/26/1	0.655	7
Selvaraj, 2010	India	South Asian	PTB	203/203	150/49/4	152/46/5	0.502	8
Wang, 2017	PR China	East Asian	TB	310/622	177/111/22	371/216/35	0.631	7
Wu, 2015	PR China	East Asian	TB	334/422	253/75/6	342/76/4	0.922	8
*TLR6 rs5743810*					TT/TC/CC			
Barletta-Naveca, 2018	Brazil	Mixed	PTB	242/174	176/58/8	120/50/4	0.649	7
Ma, 2007	USA	African	TB	339/194	289/47/3	137/50/7	0.370	7
Ma, 2007	USA	Caucasian	TB	373/114	291/72/10	78/31/5	0.404	7
Selvaraj, 2010	India	South Asian	PTB	199/202	197/2/0	199/3/0	0.915	8
Sinha, 2014	India	South Asian	PTB	204/124	196/8/0	119/5/0	0.819	7
Wu, 2015	PR China	East Asian	TB	334/422	321/13/0	410/12/0	0.767	8
*TLR8 rs3764879*					CC/CG/GG			
Dalgic, 2011	Turkey	Caucasian	PTB	124/150	36/62/26	41/85/24	0.070	8
Davila, 2008	Singapore	East Asian	PTB	140/152	78/48/14	87/56/9	0.998	7
Salie, 2015	South Africa	African	TB	220/334	90/96/34	154/144/36	0.788	7
*TLR8 rs3764880*					AA/AG/GG			
Dalgic, 2011	Turkey	Caucasian	PTB	62/78	23/26/13	37/26/15	0.014	8
Davila, 2008	Singapore	East Asian	PTB	140/152	78/48/14	87/56/9	0.998	7
Kobayashi, 2012	Indonesia	South Asian	PTB	527/555	342/92/93	348/119/88	<0.001	8
Salie, 2015	South Africa	African	TB	199/306	82/85/32	144/128/34	0.492	7
Wang, 2018	PR China	East Asian	PTB	285/304	203/76/6	209/82/13	0.181	7
*TLR9 rs187084*					AA/AG/GG			
Barletta-Naveca, 2018	Brazil	Mixed	PTB	192/192	67/102/23	84/88/20	0.665	7
Jahantigh, 2013	Iran	South Asian	PTB	124/149	63/51/10	82/59/8	0.532	8
Olesen, 2007	Gambia	African	PTB	318/339	171/122/25	186/132/21	0.705	8
Selvaraj, 2010	India	South Asian	PTB	193/218	75/91/27	84/92/32	0.228	8
Wang, 2018	PR China	East Asian	PTB	789/807	313/360/116	339/364/104	0.684	7
*TLR9 rs352139*					GG/GA/AA			
Kobayashi, 2012	Indonesia	South Asian	PTB	537/560	199/279/59	259/233/68	0.168	8
Salie, 2015	South Africa	African	TB	427/440	175/195/57	159/209/72	0.812	7
Varzari, 2019	Germany	Caucasian	PTB	119/234	49/69/12	61/126/47	0.217	7
Yang, 2013	PR China	East Asian	PTB	397/196	137/195/65	68/95/33	0.985	7
*TLR9 rs5743836*					AA/AG/GG			
Barletta-Naveca 2018	Brazil	Mixed	PTB	193/192	141/45/7	127/63/2	0.054	7
Mittal, 2018	India	South Asian	PTB	233/143	184/47/2	121/20/2	0.280	7
Olesen, 2007	Gambia	African	PTB	320/342	104/154/62	101/175/66	0.527	8
Salie, 2015	South Africa	African	TB	431/435	147/191/93	176/184/75	0.027	7
Selvaraj, 2010	India	South Asian	PTB	198/201	168/29/1	167/32/2	0.737	8
Torres-García, 2013	Mexico	Mixed	PTB	90/90	82/8/0	78/12/0	0.498	8
Wu, 2015	PR China	East Asian	TB	334/422	141/174/19	216/181/25	0.105	8

TB: tuberculosis; PTB: pulmonary tuberculosis; EPTB: extrapulmonary tuberculosis; HWE: Hardy–Weinberg equilibrium; NOS: Newcastle–Ottawa scale; NA: not available.

### TLR gene polymorphisms and TB

The results of overall and subgroup analyses are summarised in Table 2. The combined analyses showed that *TLR1* rs4833095 (recessive model: *P* = 0.02, OR = 1.17, 95% CI 1.03–1.33), *TLR1* rs5743557 (dominant model: *P* < 0.0001, OR = 1.34, 95% CI 1.17–1.54; over-dominant model: *P* = 0.02, OR = 0.85, 95% CI 0.75–0.97; allele model: *P* = 0.04, OR = 1.19, 95% CI 1.01–1.41), *TLR1* rs5743596 (dominant model: *P* = 0.01, OR = 1.18, 95% CI 1.04–1.35; over-dominant model: *P* = 0.02, OR = 0.86, 95% CI 0.75–0.98), *TLR2* rs3804099 (dominant model: *P* = 0.002, OR = 1.16, 95% CI 1.06–1.28; over-dominant model: *P* = 0.0002, OR = 0.83, 95% CI 0.76–0.92), *TLR2* rs5743704 (dominant model: *P* = 0.01, OR = 0.49, 95% CI 0.29–0.84; over-dominant model: *P* = 0.01, OR = 2.02, 95% CI 1.19–3.45; allele model: *P* = 0.01, OR = 0.50, 95% CI 0.29–0.85), *TLR2* rs5743708 (dominant model: *P* < 0.0001, OR = 0.37, 95% CI 0.24–0.55; over-dominant model: *P* < 0.0001, OR = 2.74, 95% CI 1.81–4.13; allele model: *P* < 0.0001, OR = 0.38, 95% CI 0.25–0.57), *TLR6* rs5743810 (dominant model: *P* = 0.0005, OR = 1.52, 95% CI 1.20–1.91; over-dominant model: *P* = 0.002, OR = 0.68, 95% CI 0.53–0.87) and *TLR8* rs3764879 (recessive model: *P* = 0.02, OR = 1.51, 95% CI 1.06–2.16) polymorphisms were significantly associated with susceptibility to TB in overall population. Further subgroup analyses revealed similar significant findings for *TLR1* rs4833095, *TLR1* rs5743557, *TLR1* rs5743596, *TLR1* rs5743618, *TLR2* rs3804099, *TLR2* rs5743704, *TLR2* rs5743708, *TLR4* rs4986790 and *TLR4* rs4986791 polymorphisms in certain ethnicities (see Table 2).

**Table 2.** Meta-analysis results on associations between *TLR* gene polymorphisms and TB in different genetic models.

Polymorphisms	Population	Sample size, case/control	Dominant comparison		Recessive comparison		Over-dominant comparison		Allele comparison	
			P-value	OR (95% CI)	P-value	OR (95% CI)	P-value	OR (95% CI)	P-value	OR (95% CI)
*TLR1 rs4833095*	Overall	4170/3333	0.73	1.03 (0.87–1.22)	**0.02**	1.17 (1.03–1.33)	0.08	0.87 (0.74–1.02)	0.51	0.96 (0.86–1.07)
	Caucasian	761/463	**0.002**	**0.67 (0.52–0.86)**	**0.006**	**1.54 (1.13–2.10)**	0.47	1.09 (0.86–1.40)	**0.0002**	**0.71 (0.60–0.85)**
	East Asian	2008/1648	0.11	1.12 (0.98–1.28)	0.55	1.06 (0.88–1.26)	0.13	0.85 (0.69–1.05)	0.42	1.04 (0.95–1.14)
	South Asian	738/684	0.35	0.90 (0.72–1.12)	0.34	1.36 (0.72–2.55)	0.60	0.83 (0.41–1.66)	0.17	0.90 (0.77–1.05)
*TLR1 rs5743557*	Overall	2008/1647	**< 0.0001**	**1.34 (1.17–1.54)**	0.37	0.84 (1.57–1.23)	**0.02**	**0.85 (0.75–0.97)**	**0.04**	**1.19 (1.01–1.41)**
	East Asian	2008/1647	**< 0.0001**	**1.34 (1.17–1.54)**	0.37	0.84 (1.57–1.23)	**0.02**	**0.85 (0.75–0.97)**	**0.04**	**1.19 (1.01–1.41)**
*TLR1 rs5743596*	Overall	2008/1647	**0.01**	**1.18 (1.04–1.35)**	0.69	0.96 (0.77–1.19)	**0.02**	**0.86 (0.75–0.98)**	0.22	1.10 (0.94–1.29)
	East Asian	2008/1647	**0.01**	**1.18 (1.04–1.35)**	0.69	0.96 (0.77–1.19)	**0.02**	**0.86 (0.75–0.98)**	0.22	1.10 (0.94–1.29)
*TLR1 rs5743604*	Overall	1896/1730	0.60	0.93 (0.71–1.21)	0.22	1.10 (0.94–1.28)	0.92	0.99 (0.81–1.21)	0.10	0.93 (0.84–1.02)
	East Asian	1362/1172	0.93	0.98 (0.67–1.44)	0.06	1.20 (0.99–1.44)	0.32	0.92 (0.79–1.08)	0.55	0.94 (0.77–1.15)
*TLR1 rs5743618*	Overall	3446/3014	0.68	1.08 (0.76–1.53)	0.33	0.71 (0.35–1.43)	0.65	0.93 (0.67–1.28)	0.70	1.07 (0.77–1.48)
	Caucasian	745/416	0.67	1.19 (0.55–2.58)	0.94	0.94 (0.20–4.35)	**0.02**	**0.73 (0.57–0.95)**	0.83	1.10 (0.45–2.69)
	East Asian	1217/1332	0.82	0.91 (0.40–2.06)	0.55	1.58 (0.35–7.04)	0.86	1.08 (0.46–2.54)	0.77	0.89 (0.43–1.88)
	South Asian	565/532	0.90	0.92 (0.28–3.02)	0.91	0.89 (0.13–6.15)	0.92	1.07 (0.32–3.53)	0.88	0.92 (0.31–2.73)
*TLR2 rs3804099*	Overall	3585/4308	**0.002**	**1.16 (1.06–1.28)**	0.08	1.11 (0.99–1.25)	**0.0002**	**0.83 (0.76–0.92)**	0.13	1.09 (0.98–1.21)
	Caucasian	555/449	0.20	1.39 (0.84–2.29)	0.97	0.99 (0.48–2.02)	**0.01**	**0.72 (0.55–0.92)**	0.48	1.17 (0.76–1.79)
	East Asian	1196/1865	0.08	1.14 (0.98–1.32)	0.08	1.14 (0.98–1.32)	**0.04**	**0.84 (0.71–0.99)**	0.05	1.12 (1.00–1.25)
*TLR2 rs3804100*	Overall	2217/2037	0.60	1.07 (0.82–1.40)	0.73	1.12 (0.60–2.07)	0.19	0.91 (0.79–1.05)	0.73	1.05 (0.79–1.39)
	East Asian	1152/1081	0.69	0.93 (0.65–1.33)	0.45	1.31 (0.65–2.61)	0.79	0.98 (0.82–1.16)	0.62	0.91 (0.63–1.32)
*TLR2 rs5743704*	Overall	1479/1168	**0.01**	**0.49 (0.29–0.84)**	NA	NA	**0.01**	**2.02 (1.19–3.45)**	**0.01**	**0.50 (0.29–0.85)**
	South Asian	472/472	**0.009**	**0.14 (0.03–0.61)**	NA	NA	**0.009**	**7.18 (1.63–31.70)**	**0.01**	**0.14 (0.02–0.62)**
*TLR2 rs5743708*	Overall	3062/2811	**< 0.0001**	**0.37 (0.24–0.55)**	NA	NA	**< 0.0001**	**2.74 (1.81–4.13)**	**< 0.0001**	**0.38 (0.25–0.57)**
	Caucasian	301/305	**< 0.0001**	**0.27 (0.14–0.49)**	NA	NA	**< 0.0001**	**3.77 (2.04–6.97)**	**< 0.0001**	**0.29 (0.16–0.53)**
	South Asian	1189/992	0.61	0.80 (0.34–1.88)	NA	NA	0.61	1.25 (0.53–2.93)	0.61	0.80 (0.34–1.88)
*TLR4 rs4986790*	Overall	4042/4053	0.09	0.89 (0.79–1.02)	0.18	1.32 (0.88–1.96)	0.18	1.09 (0.96–1.24)	0.05	0.89 (0.80–1.00)
	Caucasian	886/751	0.36	1.19 (0.82–1.73)	0.52	0.40 (0.03–6.46)	0.40	0.85 (0.58–1.24)	0.33	1.20 (0.83–1.72)
	East Asian	644/1044	**0.03**	**0.79 (0.63–0.98)**	0.18	1.40 (0.86–2.28)	0.11	1.20 (0.96–1.50)	**0.02**	**0.81 (0.67–0.97)**
	South Asian	821/821	0.30	0.70 (0.36–1.38)	0.47	3.94 (0.10–60.02)	0.36	1.31 (0.73–2.35)	0.29	0.69 (0.34–1.38)
	African	1075/818	0.56	1.08 (0.84–1.39)	0.12	0.34 (0.09–1.34)	0.81	0.97 (0.75–1.26)	**0.04**	**1.28 (1.01–1.63)**
*TLR4 rs4986791*	Overall	3268/3085	0.17	0.90 (0.78–1.05)	0.22	1.30 (0.85–1.99)	0.34	1.08 (0.92–1.26)	0.11	0.90 (0.78–1.02)
	Caucasian	368/294	**0.05**	**2.05 (1.01–4.14)**	NA	NA	**0.05**	**0.49 (0.24–0.99)**	0.05	2.00 (1.00–4.01)
	East Asian	644/1044	0.09	0.83 (0.67–1.03)	0.22	1.37 (0.83–2.26)	0.23	1.15 (0.92–1.43)	0.06	0.84 (0.70–1.01)
	South Asian	821/820	0.21	0.71 (0.42–1.21)	0.11	2.91 (0.78–10.93)	0.31	1.19 (0.85–1.68)	0.16	0.69 (0.41–1.16)
	African	778/486	0.10	1.50 (0.92–2.44)	0.36	0.22 (0.01–5.45)	0.13	0.69 (0.42–1.12)	0.08	1.53 (0.95–2.46)
*TLR6 rs5743810*	Overall	1691/1230	**0.0005**	**1.52 (1.20–1.91)**	0.18	0.63 (0.32–1.23)	**0.002**	**0.68 (0.53–0.87)**	0.08	1.38 (0.96–1.98)
	South Asian	403/326	0.78	1.15 (0.44–2.98)	NA	NA	0.78	0.87 (0.34–2.27)	0.78	1.14 (0.44–2.95)
*TLR8 rs3764879*	Overall	484/636	0.39	0.90 (0.70–1.15)	**0.02**	**1.51 (1.06–2.16)**	0.48	0.92 (0.72–1.17)	0.08	0.85 (0.71–1.02)
*TLR8 rs3764880*	Overall	1213/1395	0.86	0.99 (0.84–1.16)	0.17	1.18 (0.93–1.50)	0.42	0.93 (0.78–1.11)	0.39	0.95 (0.84–1.07)
	East Asian	425/456	0.72	1.05 (0.80–1.39)	0.93	0.94 (0.26–3.36)	0.73	0.95 (0.71–1.27)	0.74	1.04 (0.82–1.32)
*TLR9 rs187084*	Overall	1616/1705	0.12	0.90 (0.78–1.03)	0.17	1.16 (0.94–1.44)	0.32	1.07 (0.93–1.23)	0.12	0.92 (0.83–1.02)
	South Asian	317/367	0.69	0.94 (0.69–1.28)	0.78	1.07 (0.66–1.72)	0.34	1.16 (0.85–1.57)	0.90	1.01 (0.81–1.27)
*TLR9 rs352139*	Overall	1480/1430	0.64	1.10 (0.73–1.66)	0.05	0.80 (0.65–1.00)	0.27	1.15 (0.89–1.48)	0.33	1.17 (0.85–1.62)
*TLR9 rs5743836*	Overall	1799/1825	0.62	0.94 (0.75–1.19)	0.22	1.15 (0.92–1.45)	0.92	1.01 (0.79–1.29)	0.09	0.91 (0.82–1.01)
	South Asian	431/344	0.53	0.89 (0.61–1.30)	0.46	0.56 (0.12–2.58)	0.41	1.18 (0.80–1.74)	0.68	0.93 (0.65–1.32)
	African	751/777	0.71	0.93 (0.62–1.38)	0.22	1.17 (0.91–1.51)	0.95	0.99 (0.81–1.21)	0.54	0.92 (0.70–1.21)

Values in bold indicate a statistically significant difference between cases and controls.

OR: odds ratio; CI: confidence interval.

### Sensitivity analyses

We performed sensitivity analyses by deleting one study at a time to test the effects of individual studies on combined results. No altered results were observed in overall and subgroup comparisons, which indicated that our findings were statistically robust.

### Publication biases

We used funnel plots to assess publication biases. We did not find obvious asymmetry of funnel plots in any comparisons, which suggested that our findings were unlikely to be impacted by severe publication biases.

## Discussion

TLRs, a group of PRRs for structural conserved exogenous protospacer adjacent motifs, play vital roles in evoking immune reactions in response to infectious stimuli.^5,6^ The interaction of TLRs with their corresponding ligands activates the TLR signalling pathway, which leads to pro-inflammatory cytokine production and leucocyte infiltration.^7,8^ Given the crucial roles of TLRs in regulating immune responses against exogenous pathogens, the potential associations of certain *TLR* gene polymorphisms with susceptibility to infectious diseases such as TB were extensively studied, but the results of these studies were contradictory. Therefore, we performed the present meta-analysis of all published genetic association studies on the relationship between *TLR* gene polymorphisms and TB in order to obtain a more conclusive result.

To our knowledge, this is the most comprehensive meta-analysis to date on associations between *TLR* gene polymorphisms and TB, and our combined analyses suggested that *TLR1* rs4833095, *TLR1* rs5743557, *TLR1* rs5743596, *TLR1* rs5743618, *TLR2* rs3804099, *TLR2* rs5743704, *TLR2* rs5743708, *TLR4* rs4986790, *TLR4* rs4986791, *TLR6* rs5743810 and *TLR8* rs3764879 polymorphisms were all significantly associated with TB in certain ethnicities. The stabilities of synthetic results were evaluated by sensitivity analyses, and no alterations of results were observed in any comparisons, which suggested that our findings were statistically robust.

As for evaluation of heterogeneities, we found that for *TLR1* rs4506565, *TLR4* rs4986790, *TLR4* rs4986791 and *TLR9* rs5743836 polymorphisms, significant heterogeneities existed among the included studies. Thus, most of the combined analyses for these polymorphisms were performed with REMs. However, in further subgroup analyses, an obvious reduction tendency of heterogeneity was found in both Asians and Caucasians, which suggested that differences in ethnic background could largely explain observed heterogeneities between studies. The obvious heterogeneities that existed between included studies for *TLR1* rs4506565, *TLR4* rs4986790, *TLR4* rs4986791 and *TLR9* rs5743836 polymorphisms in the overall analyses also indicated that the distributions of these *TLR* polymorphisms vary greatly from population to population. Therefore, the genetic associations between these *TLR* polymorphisms and TB may be ethnicity specific, and we should not generalise these results to a broader population.

Several factors need to be pointed out about the current study. First, the exact underlying molecular mechanisms of our positive findings remains to be explored by experimental studies, but we speculate that investigated polymorphisms of the *TLR* gene may lead to alterations in gene expression or changes in protein structure, which may subsequently affect the biological functions of the TLR signalling pathway and, ultimately, individual susceptibility to TB. Second, the pathogenic mechanism of TB is extremely complex, and hence despite our positive findings, it is unlikely that a single gene polymorphism could significantly contribute to its development. Thus, we strongly recommend further studies to perform haplotype analyses and explore potential gene–gene interactions.^12,13^ Third, to measure the effects of certain genetic factors on disease occurrence and development more precisely, gene–environment interactions should also be considered. However, since the included studies only focused on the effects of *TLR* gene polymorphisms on individual susceptibility to TB, such analyses were not applicable in the current meta-analysis.^14^ Fourth, the present meta-analysis aimed to explore associations between all *TLR* gene polymorphisms and TB. However, only 17 polymorphisms could be analysed in the current study because no other *TLR* polymorphisms were investigated by at least two different genetic association studies. Fifth, it should be noted that a recent meta-analyses conducted by Schurz et al.^15^ also tried to explore potential associations between *TLR1*, *TLR 2*, *TLR4*, *TLR6* and *TLR9* variants and TB. However, many related studies have been published in the last three yr. Therefore, an updated meta-analysis is warranted. The sample sizes of our analyses were also significantly larger than that of the previous meta-analysis, which could significantly reduce the risk of obtaining false-positive or false-negative results. So, our work should be considered as a valuable supplementary work to the existing literature.

This meta-analysis also has some limitations. First, although the methodology qualities of the included studies were generally good, it should be noted that we did not have direct access to genotypic distributions of investigated polymorphisms according to the base characteristics of the study subjects. Therefore, our results were derived from unadjusted estimations, and failure to conduct further adjusted analyses for baseline characteristics of participants such as age, sex and co-morbidity conditions may influence the reliability of our findings.^16,17^ Second, significant heterogeneities were detected in certain subgroup comparisons, which indicated that the inconsistent results of the included studies could not be fully explained by differences in ethnic background, and other unmeasured characteristics of participants may also partially attribute to between-study heterogeneities.^18^ Third, since only published articles were eligible for analyses, although funnel plots revealed no obvious publication biases, we still could not rule out the possibility of potential publication biases.^19^ Taken these limitations into consideration, the results of the current study should be interpreted with caution.

In conclusion, the present meta-analysis indicated that *TLR1* rs4833095, *TLR1* rs5743557, *TLR1* rs5743596, *TLR1* rs5743618, *TLR2* rs3804099, *TLR2* rs5743704, *TLR2* rs5743708, *TLR4* rs4986790, *TLR4* rs4986791, *TLR6* rs5743810 and *TLR8* rs3764879 polymorphisms were all significantly associated with TB in certain ethnicities. These results suggest that these polymorphisms may be used to identify individuals at high risk of developing TB. The exact underlying molecular mechanisms of our positive findings remain to be explored by future experimental studies, but we speculate that these *TLR* polymorphisms may lead to alterations in gene expression or changes in TLR protein structure, which may subsequently affect biological activities of TLR, impact immune responses against exogenous pathogens and ultimately alter individual susceptibility to TB. Moreover, it is worth noting that many genetic comparisons in the current study were only based on a limited number of studies. So, further well-designed studies are still warranted to confirm our findings.

## Supplemental Material

Supplemental material for Associations between genetic polymorphisms of TLRs and susceptibility to tuberculosis: A meta-analysis Click here for additional data file. Supplemental Material for Associations between genetic polymorphisms of TLRs and susceptibility to tuberculosis: A meta-analysis by Yong Zhou and Mengtao Zhang in Innate Immunity
