# Supplementary figures and images for: Associations between genetic polymorphisms of TLRs and susceptibility to tuberculosis: A meta-analysis
Source: Innate Immun. 2019 Jul 18;26(2):75–83. doi: 10.1177/1753425919862354 (PMC7016404; doi:10.1177/1753425919862354)

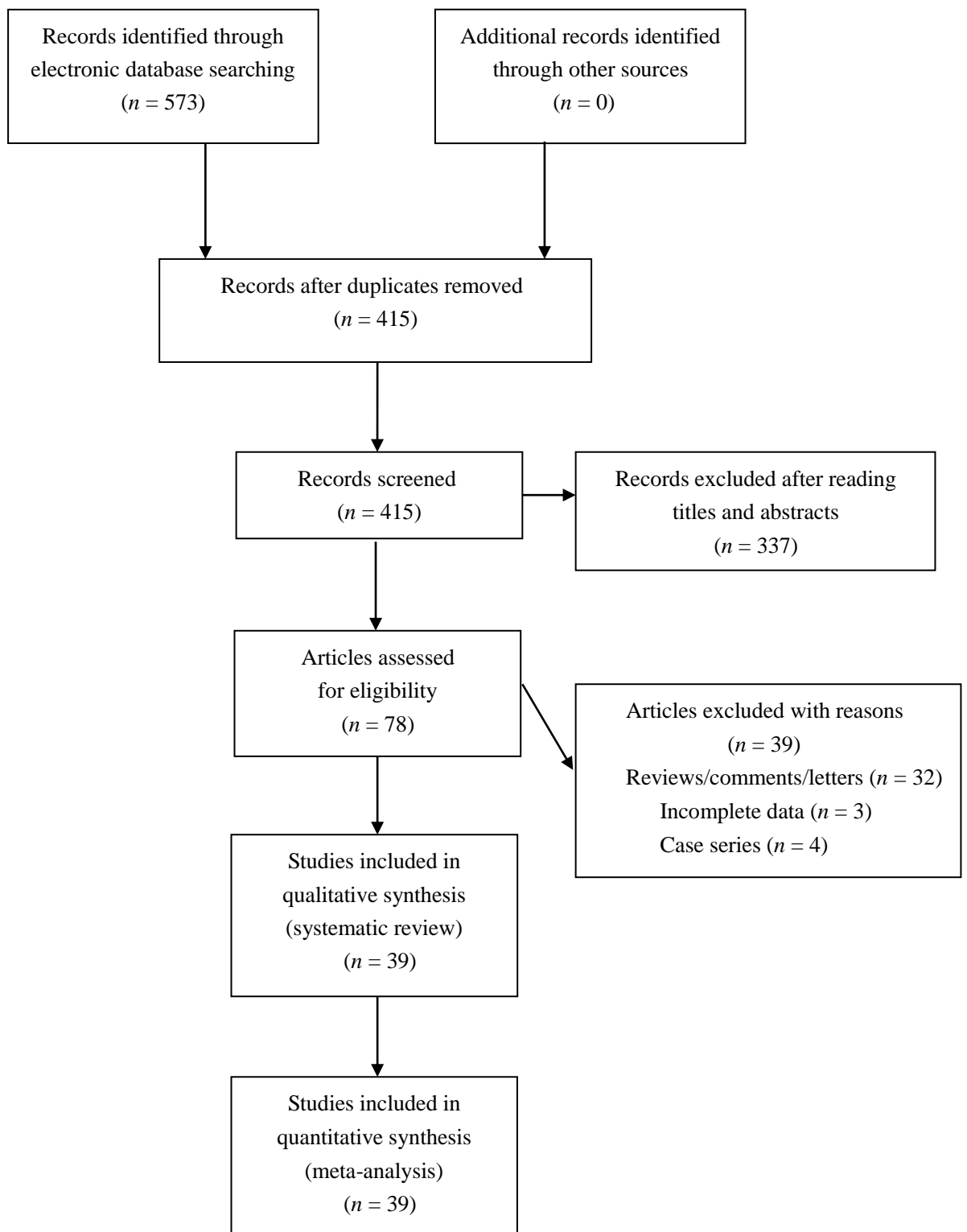

**Supplementary Figure 1.** Flowchart of study selection for the present study.

Supplement: Supplemental material for Associations between genetic polymorphisms of TLRs and susceptibility to tuberculosis: A meta-analysis [file Supplemental_Material1.pdf]
